# Toxicological qualities and detoxification trends of fruit by-products for valorization: A review

**DOI:** 10.1515/biol-2025-1105

**Published:** 2025-05-21

**Authors:** Zenebe Tadesse Tsegay, Slim Smaoui, Theodoros Varzakas

**Affiliations:** Department of Food Science and Post-Harvest Technology, College of Dryland Agriculture and Natural Resources, Mekelle University, P.O. Box 231, Mekelle, Ethiopia; Laboratory of Microbial and Enzymatic Biotechnologies and Biomolecules, Center of Biotechnology of Sfax (CBS), University of Sfax, Road of Sidi Mansour Km 6, P.O. Box 1177, Sfax, 3018, Tunisia; Department of Food Science and Technology, University of the Peloponnese, Antikalamos, 24100, Kalamata, Greece

**Keywords:** toxicology, qualities, detoxification, fruit, by-products, valorization

## Abstract

The abundant and renewable resources from fruit by-products are getting emphasis on their valorization. These by-products may contain toxic substances due to factors such as cultivation, harvesting, transportation, preservation, or processing. Hence, presenting scientific overviews of the toxicological qualities and detoxification trends of these by-products are critical for implicating their possible valorization. The present demand for valorization of fruit by-products requires emphasis and methodologies for the detoxification of any toxicants to develop healthier products. This review emphasized the toxicological qualities of by-products from fruits for which the maximum global production occurred in 2022. In this review, heavy metals (arsenic, cadmium, cobalt, chromium, nickel, lead, and mercury), mycotoxins, toxicant organic compounds, anti-nutritional factors, and pesticide/fungicide residues of the selected fruit byproducts were discussed. Current trends to reduce possible toxicants of these by-products during their valorization were emphasized. Novel functional foods valorized from these fruit by-products and future perspectives of detoxification were also focused on in this review.

## Graphical abstract

**Figure:**
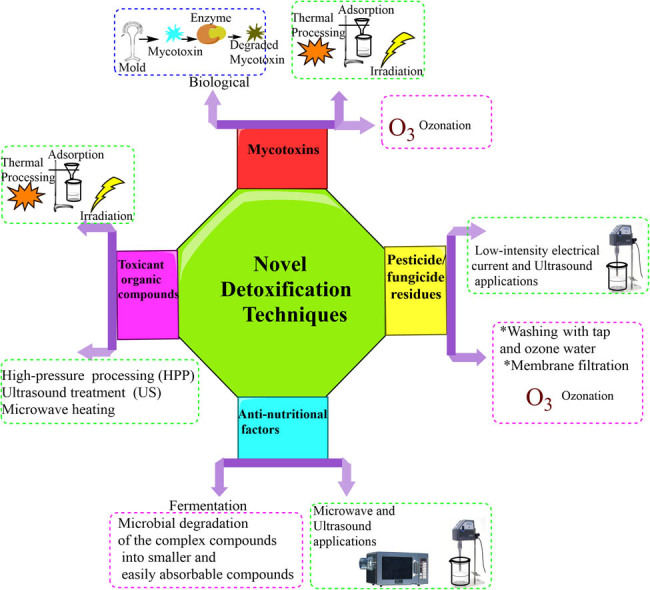

## 1 Introduction

The global population is growing rapidly, which demands more resources. Alongside the expanding growth of fruit production, optimum utilization mechanisms of their by-products are very important. Valorizing fruit by-products means employing and ensuring methods to exploit fruit wastes such as skin, pomace, and seeds as excellent raw materials for small scale and industrial based usage. Toxicants are biological and chemical components present in fruit by-products that can be harmful for human consumption when their amount is beyond the permissible level. Improving the valorization of fruit by-products not only ensures proper utilization of agricultural products but also protects environmental pollution. However, some toxic components may be available in these fruit by-products. Sufficient data on their toxicity should be available for every fruit by-product. In case of available toxicant component, proper reduction mechanism are required before valorization.

Our current living style, food processing method adopted, nature of food sources, and food market globalization are susceptible to food poisoning. Therefore, many organizations such as the food and drug administration (FDA), Food Safety Commission of Japan, European Food Safety Authority, and World Health Organization (WHO) from different countries have been developing regulations on food toxicological qualities. Food poisoning agents in fruit by-products are chemical and natural toxins, which can originate from factors associated with the raw material of the food products, microorganisms, processing, and conservation conditions. Toxicants from excessive heavy metals, nitrates, oxalates, mycotoxins, and pesticide residues are the main sources of health hazards. Consumption of foods containing maximum concentrations of toxins leads to health hazards. Hence, food supplements, cosmetics, and pharmaceuticals produced from fruit by-products should ensure high food quality and safety levels.

Agro-food-by-products are susceptible to toxicants during maturation, harvesting, preservation, and processing. Although agro-food-by-products are cheaper sources, profitable for local economies, and contain potentially functional ingredients, consumers and the food industries need details of their nutritional and toxicological qualities to be able to make use of them. Health hazards mainly mycotoxins such as aflatoxins, ochratoxin A, patulin, zearalenone (ZEN), and deoxynivalenol (DON) present in fruits and its products cause health effects of mutagenic, neurotoxic, nephrotoxic, hepatic carcinoma, and immunodeficiency, respectively [1]. To minimize the risk of mycotoxin contamination, storing fruit by-products properly and discarding any visibly moldy or spoiled produce is important. Toxic heavy metals such as lead, cadmium, chromium, cobalt, and nickel can cause harmful damage to humans. These heavy metals excessively build up in edible plant and fruit bodies from the ecosystem where they are cultivated such as from downstream of leather factories (which use chromium and other chemicals). Besides, the range of hazard quotient for As, Cd, Cr, Co, Cu, Ni, Hg, and Pb should be determined [2,3]. Another potentially toxic component in fruit by-products is pesticide residue. To reduce pesticide exposure, it is advisable to wash the fruit thoroughly before consumption and consider purchasing organic produce whenever possible. Certain fruits also contain naturally occurring toxic compounds that can be harmful if consumed excessively [4,5]. For example, some nightshade fruits contain solanine, a glycoalkaloid that acts as a natural defense mechanism against pests and diseases [6,7]. Oxalates are another toxic component that can be present in fruit byproducts. Oxalates are naturally occurring substances found in many plants. Hence, ingesting high amounts of oxalates can lead to the formation of kidney stones in susceptible individuals [8]. Nitrites are an increased risk of cancer and other health issues. To minimize nitrite formation, storing these fruit by-products properly (refrigerated) and avoiding overheating them is recommended. Toxicological and anti-nutrient qualities of many fruit by-products have not been given much attention, and most of the time these parts are discarded with their hidden food, nutraceuticals, and pharmaceutical potential not realized. Hence, evaluating the toxicological and anti-nutrient contents of these by-products so that the knowledge derived can be used to encourage the use of the by-products, while also encouraging adequate consumption of fruits, is a value-added application that this review tries to address.

Liu et al. [9] applied shell thickness-dependent Raman enhancement of silver-coated gold nanoparticles to measure pesticide residues in apple, grape, pear, peach, and mango fruit peels. They reported that pesticide residues named as thiram (1.46–7.23 ng/cm^2^), chlorpyrifos (0.14–0.7 μg/cm^2^), and methyl parathion (0.025–0.5 μg/cm^2^) were found in each of the fruit peels, in which the maximum thiram (7.23 ng/cm^2^) was observed in mango peel.

Mycotoxins, toxic heavy metals such as arsenic (As), lead (Pb), cadmium (Cd), chromium (Cr), cobalt (Co), and nickel (Ni), pesticide residues, oxalates, and nitrate have recommended daily intake (RDI). Excessive presence of minerals (heavy minerals), saponin, alkaloid, hydrocyanic acid, oxalate, tannins, phytates, and nitrates in fruit by-products compared to recommended daily allowance (RDI) are considered as toxicant components. The range of hazard quotient (mg/kg) for As, Cd, Cr, Co, Cu, Ni, and Pb are 2.71–11.38, 0.60–3.32, 0.81–3.18, 0.03–0.09, 0.09–0.26, 0.08–0.34, and 0.83–2.23, respectively [2]. The acceptable daily intake (ADI) limit or toxicity level of dietary nitrates is 3.7 mg/kg body weight according to the regulations of the Joint Expert Committee of Food and Agriculture and the European Commission’s Scientific Committee on Food [10]. RDI values of potassium, magnesium, iron, zinc, manganese, copper, phosphorus, and calcium for adults are 4.6 g/day, 260 mg/day, 14 mg/day, 7 mg/day, 2.3 mg/day, 900 µg/day, 700 mg/day, and 1,000 mg/day, respectively [11]. Heavy metals like As, Pb, and Cd are considered toxicants if their concentrations are greater than the recommended level. Based on the WHO [12] recommendation, the monthly intake level should be <0.2 mg/100 g for aluminum; 0.21 mg/100 g for As and Pb, and 0.25 mg/100 g for Cd. Moreover, the maximum Pb limit for edible parts of crops for human health is 0.02 mg/100 g. Pineapple skin contained lead beyond this maximum limit (0.64 mg/100 g analyzed in dry base), whereas in fruit by-products such as orange peel, watermelon rind, banana peel, apple pomace, strawberry pomace, grape pomace, lead contents were insignificantly detected (below 0.1 µg/L) [13,14]. According to Oyeyinka and Afolayan [15], fresh banana (*M. sinensis*) peel contains a lower alkaloid (0.66 g/100 g), oxalate (37.0 g/100 g), phytate (2.78 g/100 g), and saponin (6.57 g/100 g) than boiled peel extract of the fruit that composed alkaloid (1.76 g/100 g), oxalate (40.2 g/100 g), and saponin (8.12 g/100 g). Except for oxalate, the alkaloid, phytate, and saponin in this study report are in a safe level for human consumption of the fruit peel. According to the study conducted on the local fruits in Incheon, Korea, the most frequently detected pesticide residues were chlorfenapyr, procymidone, etofenprox, pendimethalin, fluopyram, and azoxystrobin [7].

Anti-nutritional factors (ANFs) such as oxalates, hydrogen cyanides (HCN), alkaloids, phytates, tannins, and glycosides can be found in food products [16]. In particular, fruit by-products contain considerable amounts of ANFs although their level is different, which are summarized in Table 2. The anti-nutritional effect of oxalate (a salt formed from oxalic acid) is that it can bind to nutrients preventing their absorption. Hence, consuming foods containing high concentrations of oxalic acid can cause nutritional deficiencies and irritation of the lining of the gut. In addition to these sources, from the flavonoid groups, tannins are anti-nutrients that cause health effects. Mainly it involves in chelation of minerals like iron and zinc, which then reduce their absorption, as well as inhibit digestive enzymes, thus causing precipitate proteins [17]. Fruits and berries contain high amounts of oxalates, cyanide-inducing glycoside, amygdalin (AMG), sambunigrin, hydrocyanic acid, and toxins [18,19,20,21].

Our study tries to give a comprehensive review of the availability of heavy metals (As, Cd, Co, Cr, Ni, Pb, and Hg), mycotoxins, toxicant organic compounds, ANFs, and pesticide/fungicide residues in fruit by-products that have maximum (>1.2 million tons) global production from FAOSTAT during the year of 2022 [22]. Moreover, the current detoxification trends for the selected fruit by-products were discussed. We focused to review these selected fruit by-products since much of their by-products are being wasted even though these are important raw materials for small scale and industrial based exploitations. There is greater demand for valorization of these by-products due to their promising bioactive compound contents. Utilization of their by-products for better inspiration of future research and the discovery of biofuel, food ingredients, pharmaceutical, and cosmetic products are getting attention.

## 2 Fruits with maximum global production and their by-products

**Figure 1:**
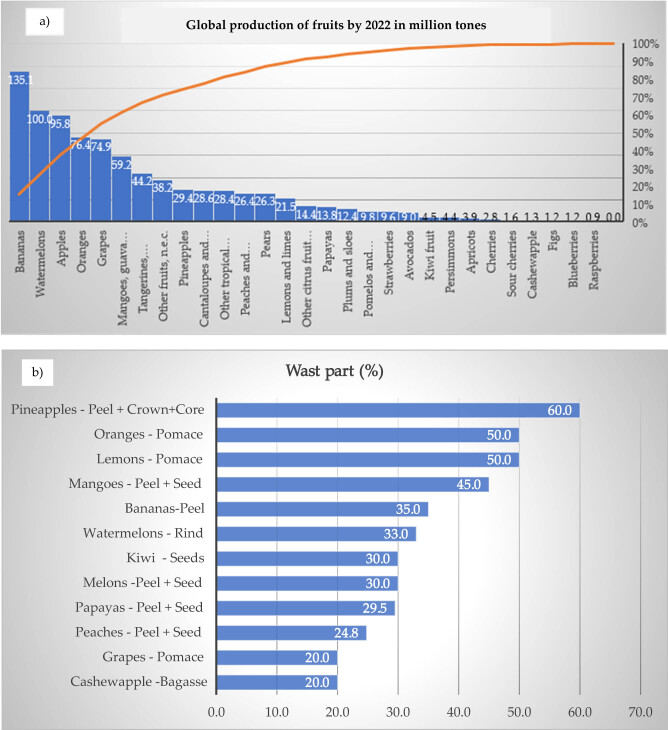
Global fruit production and their waste. (a) Global production of fruits by the year of 2022 [22]. (b) Percentage waste part of common fruits.

The global production, consumption, and generation of waste and fruits by-products are increasing from year to year. According to the FAOSTAT database, the fruits with large production during 2022 are bananas, watermelons, apples, oranges, grapes, and the remaining fruits are depicted in Figure 1a in decreasing order [22]. Many fruits are wasted during harvesting, transportation, and processing, and by-products are produced. Studies have been reporting that from the total fruit weight, about 30–50% accounts for fruit waste and by-products [23]. Percentage waste part of common fruits prioritized in the current review are depicted in Figure 1b. Nearly half of the fruit parts are discarded in terms of peels, seeds, rinds, husks, rags, roots, and pomace during the day-to-day activities in homes and agro-processing industries. It is important to consider that these fruit by-products are important plant sources containing many bioactive substances, dietary fiber, minerals, and others [24,25]. Moreover, fruit by-products contain bioactive substances that show potent ant-microbial activities [25]. Considering their maximum production and by-products that could be generated, we prioritized discussing the toxicity of the fruits with global production greater than 1.2 million tonnes.

Potential toxicants that can persist in the bioactive compounds extracted from fruit by-products are mycotoxins, pesticides, biogenic amines, heavy metals, and microbial contaminations. These contaminants are the main causes of the extracted product’s safety, such as biological instability, potential for rapid auto-oxidation, potential pathogenic contaminations, high levels of active enzymes, and high water activity [26].

Many fruit by-products contain potential contaminants such as heavy metals mycotoxin, ant-nutritional contaminants, organic contaminants (biogenic amines), and pesticides [24]. The heavy metals (As, Cd, Co, Cr, Ni, and Pb) present in the prioritized fruit by-products are summarized in Table 1. Moreover, the selected fruit by-products containing mycotoxins, toxicant organic compounds, ANFs, and pesticide/fungicide residues are presented in Table 2.

**Table 1:** Dominant heavy metals in fruit by-products

		Heavy metals (mg/kg)						References
Commodity		As	Cd	Cr	Co	Ni	Pb	
Apple	Peel		<1	0.57–3.8		<1	<1	[37,40,41]
	Seed							
	Pomace							
Apricot	Kernel		0.1–6		2.7–35.7			[42]
	Pomace							
Avocados	Seeds			0.57–2.29	0.00		0.00	[40,43]
Bananas	Peels	<0.0001	0.0013–0.18	1.42–4.04	0.4–47.2		0.0038–0.64	[36,44,45]
Blueberries	Pomace		0.011	0.242	0.08	0.592	0.73	[46]
Grape	Peel/skin/pomace		<0.5	0.18–2.41		<0.5	0.021–1.11	[34,35,37]
Lemons	Peels by-products	0.004	0.00047–0.25	1.04	0.038	0.973–1.24	0.0188–0.22	[34,36,39]
Limes	By-products	ND	0.003		0.073	1.678	0.128	[39]
Mangoes				0.33	28.0			[45]
Orange	Peel		<0.5	1.04–4.14	0.015	0.05–2.36	0.01–1.75	[34,36,37,39,47]
	Seed							
Papayas	Peel	0.0287–0.03	0.0027–0.00685	0.278–7.36	0.4–219	0.246–13.2	0.03–0.044	[45,48,49]
	Seed							
Peaches				0.17–1.38			<0.10	[50]
Pineapples	Peels	<0.0001	0.0074	8.77	70.3		0.0027	[44,45]
Plums	Peels	1.2	ND			2.8	ND	[51,52]
	Kernels	<0.1	0.13		0.2	1.7	0.13	
Pomelos	Peels		1.36 × 10^−3^				0.0296	[34]
Raspberries	Pomace		0.0084	0.116	0.073	0.762	0.047	[46]
Strawberry	Pomace		0.0078	<0.01		0.00336	0.0011	[53,54]
Mandarins			0.00062				0.020	[38]
Watermelons	Peel		0.008–0.1	4.65			0.06–0.09	[40,55]
	Seed							

ND: Not determined.

**Table 2:** Toxicant compounds in fruit by-products

Commodity		Mycotoxins (mg/kg)	Toxicant organic compounds (mg/kg)	Anti-nutritional contaminants (mg/kg)	Fungicide/pesticide residues (mg/kg)	References
Apple	Peel/skin	AOH-3-S = 1.4–10.8 × 10^−3^	Naphthaleneacetic acid = 0.433	Oxalates = 890.7	Acetamiprid = 72–81	[57,58,59,60,61]
	Seed			HCN = 960.4		
	Pomace	AME-3-S = 1.7–10 × 10^−3^	AMG = 1,000–4,000			
				Alkaloids = 79.9		
				Phytates = 14.2		
Apricots	Seeds	AFB1 and AFB2 = 0.0017–22.451	AMG = 52,000	Tannins = 1564.4		[62,63,64]
				Phytic acid = 1171.5		
				Oxalates = 156		
Avocados	Seeds			Tannins = 7.6		[43]
				Alkaloids = 54		
				Phytates = 4.4		
				Oxalates = 44		
Bananas	Peels			Phytate = 2.11–9,270	Chlorpyrifos = 0.11–0.8	[65,66]
				Alkaloids = 0.45–5.45	methiocarb = 0.014–0.183	
				Oxalate = 20–8,280		
				Glycosides 149,020		
				Tannin = 900		
Blueberries	Pomace				Chlorpyrifos-methyl = 4.27 × 10^−3^	[67,68]
					Thiametoxan 5.15 × 10^−3^	
					Azoxytrobin 0.187	
Grapes	Peel/skin	OTA = 0.1–0.32 × 10^−3^		Oxalate = 0.6–0.7	Cyprodinil = 1.07–1.94	[34,35,69]
	Pomace			Tannins = 0.274–0.41		
					Dimethomorph = 0.56–2.73	
					Feamoxadone = 1.55	
Lemons	Peels/pomace			Oxalate = 0.4–0.5	Pyriproxyfen = 0.039	[34,39,69]
				Tannins = 0.28	Fludioxonil = 0.008	
					Propiconazole = 0.008	
					Pyrimethanil = 3.8	
Limes	Pomace				Fludioxonil = 0.009	[39]
					Flutriafol = 0.11	
					Propiconazole = 0.008	
					Imazalil = 1.49	
					Tebuconazole = 0.076	
Mangoes			Putrescine = 0.9	Phytic acid = 254.8		[70,71,72]
				Oxalate = 724		
				Tannin = 153 × 10^3^		
Orange	Peel	Tryptoquialanine A = 248.1	Putrescine = 11.34–151.1	Tannins = 0.228	Etoxazole = 0.010–0.637	[34,60,69,72,73,74,75]
	Pomace	Tryptoquialanine C = 375.80		Oxalates = 1.2–997.8	Imidacloprid = 162.16	
				HCN = 397.9	Carbendazim = 372.1	
				Alkaloids = 54.4	Abamectin = 0.261	
				Phytates = 23.4	Cypermethrin = 495.6	
					Prochloraz = 8.11.7	
Papayas	Peel		Putrescine = 5.3–19.3	Tannin = 17.6–500		[71,72,76]
				Oxalate = 0.6		
				Phytate = 0.6		
Peaches	Seeds		Putrescine = 1.82–2.02	Tannins = 5137.6		[63,72]
				Phytic acid = 2126.3		
				Oxalates = 385.9		
				HCN = 372		
Pears			Putrescine = 23.6–24.2			[72]
Pineapples	Pineapple shell	Fusarium = 250	Putrescine = 1.39–7.96	Oxalates = 0.4–1290.6	Carbaryl = 0.262	[60,69,72,77,78,79]
				HCN = 715	Carbofuran = 14.3	
	Pomace	Aflatoxin B_2_ = 0.008 × 10^−3^		Alkaloids = 161.9	Fenobucarb = 0.01	
		Aflatoxin G_1_ = 0.013–0.033 × 10^−3^		Phytates = 19.9	Isoprocarb = 0.313	
		OTA = 0.051 × 10^−3^			Propachlor = 0.015	
Pomelos	Peels			Tannins = 0.315	Total pesticides = 0.216	[34]
Strawberry	Pomace		Putrescine = 2.04–6.42		Total pesticides = 2.143	[53,72,80]
					Procymidone = 0.7	
			DEHP = 0.25			
					acetamiprid = 0.212	
					Boscalid = 0.745	
			DIBP = 0.283		Carbendazim = 0.13	
			and DBP = 0.222			
Tangerines	Peels	AOH = 0.003–0.017				[39]
Watermelons	Peel			Phytate = 9,900	Dimethoate = 1,730	[60,81,82]

Studying the amounts and the toxicity level of the waste and by-products of the prioritized fruits is demanded for better valorization. In particular, bananas have the maximum global production in 2022 (Figure 1a). The banana peel represents about 35% of the total fresh harvest mass of ripe fruit, a rich source of dietary fiber, protein, essential amino acids, polyunsaturated fatty acids, antioxidant compounds, and potassium (Figure 1b) [27]. About 25–30% of the apple’s total content is represented by by-products which include seeds and peels. Moreover, nearly 20% of the original grape weight accounts for its by-products mainly pomace (skins, stems, and residual pulp) and seeds. Besides, the mango processing produces by-products that range from 40 to 60%, from which about 10–20% represents peels, 10–25% is for seeds, and 15–20% is for kernels. Citrus fruit also creates about 50–70% by-products, which represent seed (1–10%), peel (flavedo and albedo) (range 60–65%), and internal tissues (30–35%) (juice sac residues and rag) [28,29]. The industrial processing of avocados produces by-products that account for 21–30% of the total, including residues like seeds and peels. Similarly, fresh processing of pineapples creates about 35–46% by-product mainly residual pulp, peels (30–42%), stems (5% core and stem share), and a core only (10%) [28,30]. As mentioned above, the fruits contained considerable by-products, which could be utilized for different types of product development. Much of the fruit by-products have promising nutritional qualities [31]. Hence, ensuring the toxicity of these by-products for better sustainability and feasibility of valorization is required.

## 3 Toxicological qualities of citrus fruit by-products

Citrus fruits are well-known sour fruits such as oranges, lemons, limes, tangerines, and grapefruit. Czech et al. [32] classified citrus fruits like orange, pomelo (*Citrus maxima*), mandarins, lemon, and grapefruits. The by-products of citrus fruits (Rag, peel, and seeds) account for about 50% [33]. These fruit by-products can contribute to many bioactive compound extractions, sources of pectin, molasses, fibers, oils, and animal feed [31]. Mainly, the citrus fruit peels are raw materials for the functional food products development such as jams, marmalade, yogurt, crackers (a thin flat crisp biscuit), and meatballs [32].

Heavy metals mainly lead, cadmium, chromium, copper, lead, and nickel were found in orange, lemon, and grapefruit peels as well as in grape skin extracts [34,35,36]. Bożym et al. [37] analyzed heavy metals (Cd, Cr, Ni, and Pb) present in grape and orange peels as shown in Table 1. Analysis of these heavy metals revealed that their concentrations are within the permissible limits for anaerobic digestion to produce biogas.

The heavy metals reported in citrus fruit peel such as orange (Cd = 0.00049, Pb = 0.01 mg/kg), lemon (Cd = 0.00047, Pb = 0.0188 mg/kg), red grapefruit (Cd = 0.0019, Pb = 0.03 mg/kg), mandarin (Cd = 0.00062, Pb = 0.02 mg/kg), lime (Cd = 0.0265, Pb = 0.00046 mg/kg), and pomelo (Cd = 0.00136, Pb = 0.0296 mg/kg) were considered. According to the norms for heavy metals (Regulation 1275/2013 and Ordinance 10/2009), the limit for animal feed consumption for cadmium and lead is 1 and 10 mg/kg, respectively. Hence, the reported heavy metal concentrations are below these limits [38].

Mateus et al. [39] investigated heavy metal concentrations in citrus by-products mainly from orange (*Citrus sinensis*), lemon (*Citrus limon*), and lime (*Citrus aur­antifulia*) considering As, Cd, Co, Hg, Ni, and Pb. The concentrations of these heavy metals are summarized in Table 1. They reported that the Hg in lemon, orange, and lime and the As in orange and lime by-products were reported as below the detection limit.

The heavy metal (lead and cadmium) analysis conducted on citrus fruit peels (orange, pomelo, lemon, and grapefruits) as shown in Table 1 was reported by Czech et al. [34]. However, according to WHO (0.1–0.2 mg/kg fresh weight of fruit for lead and 0.05 mg/kg for cadmium), the reported results are not beyond the acceptable levels. Moreover, they reported that the peels of these fruits contained tannins (Table 2). The concentration of tannins in grape, orange, and pomelo fruit peels may limit the use of these fruit peels, which demand reduction mechanisms.

According to the European Commission [56], heavy metals and other metals of safety concern (mg/Kg) analyzed in lyophilized citrus by-products (lemon. orange, and lime) should have a maximum residual level (MRL) of As (0.02 mg/kg), Cd (0.02 mg/kg), Co (not applicable), Hg (not applicable), Ni (not applicable), and Pb (0.1 mg/kg). The citrus by-products reviewed in the present study are below this MRL.

The mineral binding anti-nutrients such as oxalates and phytic acid concentrations in citrus fruits (lemon, orange, and grapes) pomace were investigated as negligible amounts (Table 2). Hence, the pomace of these fruits can be valorized into value-added products [69]. However, the presence of tannins should be considered.

Mycotoxins such as aflatoxins (AFB1, AFB2, AFG1, and AFG2), ochratoxin A (OTA), ZEN, toxin T2 (T2), and fumonisins (FB1 and FB2) were found in orange, lemon, or lime pomace. Moreover, alternariol (AOH), and alternariol mono-methyl ether (AME) were determined in other citrus fruit by-products such as tangerine (*Citrus reticulata*) peels (range from 0.003 to 0.017 mg/kg) [39,83].

Mateus et al. [39] studied the presence of pesticide residues in fresh citrus pomace. Mainly they determined these pesticides in orange (pyriproxyfen = 0.027, fludioxonil = 0.355, imazalil = 0.007, and Pyrimethanil = 2.77 mg/kg), lemon (pyriproxyfen = 0.039, fludioxonil = 0.008, propiconazole = 0.008, and pyrimethanil = 3.8 mg/kg), and lime (fludioxonil = 0.009, flutriafol = 0.11, propiconazole = 0.008, imazalil = 1.49. and tebuconazole = 0.076 mg/kg) pomace.

Socas-Rodríguez et al. [84] studied the pesticides in citrus by-products. They reported that malathion (0.045 mg/kg) and pyriproxyfen (0.0495 mg/kg) were found dominantly in the studied citrus by-product samples. The malathion was below the MRL whereas the pyriproxyfen was found approximately near the MRL (0.05 mg/kg). However, other types of pesticides studied were below the detection limit (from 0.0085 to 0.1288 mg/kg).

Mycotoxins such as AOH and AME were found in citrus fruit by-products. In particular, AOH, a type of mycotoxin, was identified in tangerine (*Citrus reticulata*) peels [39].

According to the MRL developed by European Food Safety et al. [85] for some fungicides such as carbendazim (0.2 mg/kg), thiabendazole (7 mg/kg), imazalil (4 mg/kg), and insecticides such as λ-cyhalothrin (0.2 mg/kg), carbofuran (0.01 mg/kg), and chlorpyrifos (1.5 mg/kg); the reviewed results of the fungicides and insecticides in citrus fruits were below the MRLs.

### 3.1 Oranges

Orange fruit is the fourth globally produced fruit (Figure 1). Large amounts of orange fruits are utilized for juice production, which accounts for about 85% of total processed consumption [86]. Its by-products are peels, seeds, and pomace.

The heavy metals (Cr, Ni, and Pb) analyzed in orange fruit (*Citrus sinensis*) seeds and peels were reported as beyond the RDI set by WHO/FAO [87] (Cr = 0.05–0.2, Ni = 1.4, and Pb = 0.214 mg/day), which are summarized in Table 1 [47]. Hence, careful handling of these by-products is required when valorized in functional foods.

*Citrus aurantium* (L.) is commonly known as bitter orange, sour orange, Seville orange, bigarade orange, or marmalade orange, which is a rich source of many bioactive compounds. Oral administration of these fruit peel extracts did not cause mortality or signs of acute toxicity in mice at a 2,000 mg/kg dose. Hence, this fruit peel can extract many non-toxic phytoconstituents [88].

Essential oils (EOs) extracted from bitter orange peel were studied for their oral administration toxicity in mice from 48 h to 14 days with a concentration of 2,000 mg/kg. It is reported that any of clinical symptoms, acute toxicity, or mortality, as well as no change in food intake, behavior, or body weight were observed on the tested mice at the specified time. Other studies have also proven that oral administration of mice with a dose of 5,000 mg/kg EOs did not show any such toxicity except reducing serum total cholesterol of the mice at 10 mg/kg of EOs from these extracts [88,89].

The pesticide residues (imidacloprid, abamectin, cypermethrin, and prochloraz) in orange fruits were particularly distributed in its peels and the second most distributions were in the fruit pomace (Table 2). Washing during the fruit process can reduce the pesticide residues from 43.6 to 85.4%. The fruit pomace that passed the juice extraction process contained residues from 46.0 to 94.7% of imidacloprid, abamectin, cypermethrin, and prochloraz. However, the residue carbendazim was found to lower concentration. Hence, unless proper removal of these pesticide residues is applied valorizing the orange fruit by-products could have health effects [74].

Epicarp layer of orange fruit was found to contain tryptoquialanines A and C (Table 2). However, these concentrations were found within the range of limits of tremorgenic mycotoxins, which can cause deleterious effects on vertebrates [73].

Pesticides like imidacloprid, carbendazim, abamectin, cypermethrin, prochloraz, thiabendazole, and carbaryl, and mycotoxins such as fumonisin B1, tryptoquialanines A and C are present in peels and orange by-products [73,74,90].

The ANFs reported in orange fruit peels were oxalates (997.8 mg/kg), HCN (397.9 mg/kg), alkaloids (54.4 mg/kg), and phytates (23 mg/kg). The reported value for HCN in orange fruit peel is below the threshold value (below 3,500 mg/kg) reported as the safety limit [60].

**Figure 2:**
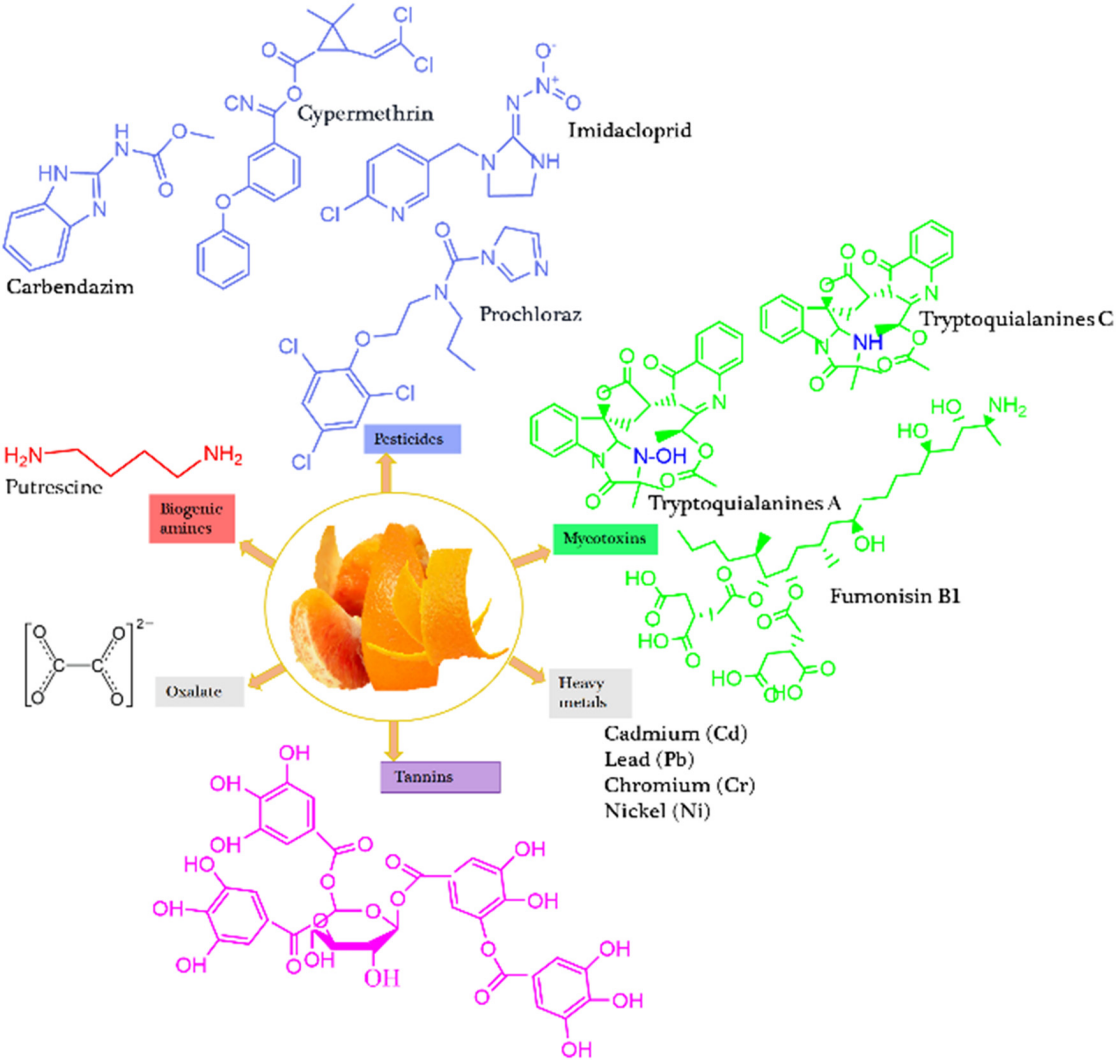
Toxicant elements and chemicals in orange fruit by-product (peel).

In general, orange fruit peels can be valorized into valuable products by applying toxicity reduction mechanisms. Figure 2 shows some toxicant components present in orange fruit peels.

### 3.2 Pomelos

Pomelos is a type of citrus fruit, that includes sweet oranges (*Citrus sinensis*), lemons (*Citrus limon*), limes (*Citrus aurantifulia*), tangerines (*Citrus reticulata*), and grapefruit (*Citrus paradisi*). About 166.4 million tonnes of these fruits and other citrus fruits (mandarins, clementines, pomelos, grape­fruits) were globally produced during 2022 [39]. About 9.6 million tonnes of pomelos were produced during 2022 (Figure 1). The pomelo peel extract accounts for 19.56% of the total fresh fruit weight [91].

Li et al. [92] analyzed the total concentration of pesticides in the pomelo waste parts (epicarp and seeds of pomelo fruit). They reported that the epicarp (0.216 mg/kg) contained the highest than the mesocarp (0.0095 mg/kg), endocarp (0.0044 mg/kg), seed (0.0038 mg/kg), and pulp (0.0011 mg/kg). The reported concentrations were below the ADI limit.

Pomelo peels contain heavy metals like Cd and Ni (Table 1), which are below the maximum level set by the European Commission [56].

Pu et al. [93] assessed pomelo seed oil cytotoxicity in human liver LO_2_ and human liver cancer Hep G2 [HEPG2] cells. They reported that IC_50_ values of the Pomelo seed oils were in the range of 16.31 and 28.8 mg/mL, which is acceptable since the non-cytotoxic compounds have IC_50_ values greater than 1 mg/mL. The cytotoxicity dose of the Majia pomelo seed oil concentration tested in human liver cancer HepG_2_ cells was in the range of 500–4,000 μg/mL, which were also found to be non-toxic.

### 3.3 Mandarin

The etoxazole concentration (0.010∼0.637 mg/kg), which is a type of pesticide in citrus (*Citrus reticulata Blanco*) peel, was found larger than its pulp part (0.010–0.011 mg/kg). However, the chronic dietary risk of etoxazole in this fruit peel was in an acceptable range (0.035–0.951%) of the daily consumption recommended dosage [75].

Rossi et al. [94] studied the cytotoxic effects of EO extracted from *Citrus deliciosa* fruit peels harvested at different stages of maturation (immature, intermediate, and mature) against HT-29 cells. They reported that the peel extract from mature fruit showed significant cytotoxic properties (IC_50_ of 110 μg/mL). Hence, bioactive compounds present in these fruit peels like monoterpenes and citronellol can kill or damage cells, and slow or stop the development of rapidly proliferating cancer cells [95].

### 3.4 Lemon

Lemon fruit structure is divided into albedo, flavedo, and pulp. The albedo and flavedo are by-products of lemon during its juice production, which are the main sources of pectin, cellulose, EOs, and pigments. Moreover, the albedo is rich in flavonoids like hesperidin and eriocitrin [96]. The heavy metal concentrations analyzed in kaffir lime peel considered were Cr, Co, Ni, Pb, Cd, Pb, and Hg as shown in Table 1. In this study, all except Hg (Hg = 0.0145 mg/kg) were reported below the detection limit [97]. However, other toxicity studies on fruit peels are scarcely studied. Hence, the valorization of the lemon fruit peel should be done with further investigations of toxicities.

### 3.5 Grape

Grapefruit is the fifth globally produced fruit with 74.9 million tons (Figure 1). The fruit skins, stems, and seeds of grapefruit account for about 20%, mostly found as pomace during grape juice processing [33].

Grape skin extract contained potential safety hazards such as pesticides (cyprodinil, dimethomorph, and feamoxadone), mycotoxins (OTA), biogenic amines (ethanolamine and ethylamine), and heavy metals (Cd and Pb) [35]. In particular, Moncalvo et al. [35] investigated the pesticide residues (cyprodinil, dimethomorph, and feamoxadone) as well as heavy metals in grape skin powders and extracts. The concentration of these pesticide residues shown in Table 2 is below the minimum recommended limit set by the European Union (cyprodinil = 15, dimethomorph = 15, and feamoxadone = 10 mg/kg). Similarly, the heavy metal concentrations are also below the minimum recommended limit (Pb = 0.23 and Cd = 0.23 mg/kg). The biogenic amines analyzed in waste grape skin powders were also found below the toxicity level. The mycotoxin (OTA = 0.1–0.32 × 10^−3^ mg/kg) content in grape skin powders was also below the maximum residue levels for ochratoxin (0.01 mg/kg). Furthermore, from the review reported by Georganas et al. [98], the potential hazards found in grape pomace were heavy metals such as As, Pb, Cd, and Ni), toxins like OTA, and biogenic amines.

**Figure 3:**
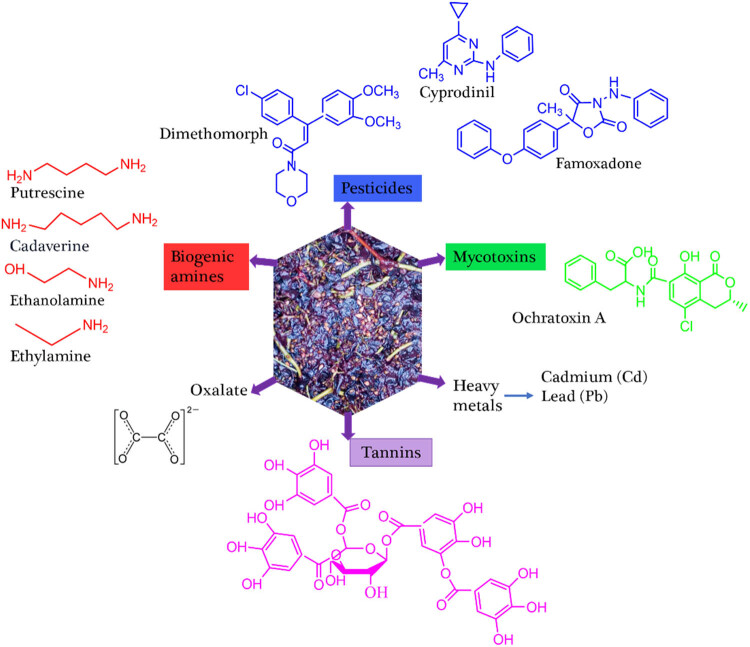
Toxicant elements and chemicals in grapefruit by-product (pomace).

Proanthocyanidins are part of polyphenols which are used as plant pigments and are found in grape seeds and skins. Proanthocyanidins found in bioactive extracts from grape seeds and skins were studied for their acute oral toxicity, genotoxicity, and lethal dose 50 (LD_50_). It is reported that their LD_50_ is greater than 5,000 mg/kg and up to 2,000 mg/kg did not show genotoxicity (micronucleated erythrocytes for 72 h treatments) [99,100]. Moreover, during daily administration of 1,420 mg polyphenols enriched with ellagitannin extracts for 4 weeks, human safety was also found safe [101]. Figure 3 depicts some toxicant components present in grapefruit pomace.

## 4 Toxicological qualities of tropical fruit by-products

Tropical fruit by-products considered here are the peel, pomace and/or seed from avocado, pineapple, banana, papaya, watermelon, and melon fruits.

Tropical fruit by-products contained hazard heavy metals. de Matuoka e Chiocchetti et al. [45] studied the heavy metals (Co and Cr) present in banana, papaya, and pineapple peels, summarized in Table 1. The amounts are above the range of hazard quotient (Cr = 0.81–3.18 mg/kg and Co = 0.03–0.09 mg/kg) [2]. Moreover, the heavy metals analyzed in pineapple and banana peels were As, Cd, Sn, Hg, and Pb. In both peels, low concentrations of these heavy metals were reported (Table 1). In particular, the As, Sn, Hg, and Pb were <1 × 10^−3^ mg/kg [44].

Hassan et al. [40] studied heavy metal concentrations in avocado seeds and watermelon silage and considered Cr and Ni concentrations. They reported that both fruit by-products contained insignificant (negligible) Ni. However, the Cr concentrations in avocado seeds and watermelon silage were 0.57–2.29 and 4.65 mg/kg.

The anti-nutritional contaminants present in avocado seeds were tannins, alkaloids, phytates, and oxalates, the reported concentrations are summarized in Table 2 [43]. Moreover, the ANFs reported in pineapple, banana, and watermelon fruit peels were oxalates, HCN, alkaloids, and phytates, and their concentrations are presented in Table 2. The reported values for HCN in these fruit peels are below the threshold value (below 3,500 mg/kg) reported as the safety limit [60].

The amount of toxic substances summarized in this review can vary from place to place and in different measurement techniques applied. Hence, the reported toxicant components imply that the by-products should be valorized with the application of further toxicant reduction techniques.

### 4.1 Avocado

Avocados have nine million tons of global production (Figure 1). Avocados contain peel (7–15%) and stone (including the seed) (14–24%) waste parts of the total fresh fruit weight. These waste parts are sources of many functional components such as antioxidants, bio-oil, bioethanol, etc. [102].

The heavy metals analysis considering Co and Pb in avocado seeds was reported as insignificant [43]. Hence, avocado fruit seeds can be valorized for many applications.

Cytotoxicity study conducted on avocado agro-industrial by-products (peel and seed) of two varieties (Hass and Fuerte) did not exhibit a cytotoxicity effect on RAW 264.7 cells. Specifically, the cytotoxicity test was performed at different concentrations ranging from 0.1 to 100 μg/mL of the fruit varieties peels and seeds extract and concentrations up to 10 μg/mL have no cytotoxicity effect [103].

Other toxicant compositions in avocado fruit by-products are scarcely studied. Hence, further toxicity studies on these by-products are required to fully valorize them.

### 4.2 Pineapple

Pineapples have about 29.4 million tons of global production. Therefore, much of its by-products can be valorized into valuable products. The pineapple fruit waste accounts for 60% of the weight of pineapple fruit interns of peeled skin, core, crown end, etc. [104].

As, Cd, Cr, Co, and Pb were found in pineapple peels (Table 1) [44,45]. The As concentration is almost insignificant, whereas the Cr and Co amounts are considerable. Hence, careful treatment is required before valorization.

The main reasons for mycotoxins development in fruit by-products are the refrigeration temperature conditions and water activity during storage. In a study on mycotoxins toxicity (aflatoxin B_2_, aflatoxin G_1,_ and OTA) levels (shown in Table 2) in pineapple shells were determined below the detection limits (2.9 × 10^−3^ mg/kg) [77].

Oxalates and phytic acid concentrations in pineapple pomace are negligible (Table 2), which assures its applicability for value-added products [69].

Stępień et al. [78] reported the pineapple skin infected with mycotoxin mainly containing fusarium (250 mg/kg). The US FDA mycotoxin exposure guidelines state that the total fusarium in cereal foods should be 2–4 mg/kg. Hence, the reported result can be hazardous to human health without proper pretreatment.

Wanwimolruk et al. [79] analyzed pesticide residues in unpeeled pineapples and reported that considering carbaryl (0.262 mg/kg), carbofuran (1.43 mg/kg), fenobucarb (0.01 mg/kg), isoprocarb (0.313 mg/kg), and propachlor (0.015 mg/kg). Moreover, they analyzed the peeled pineapples which contained pesticide residues much lower than the unpeeled.

In general, pineapple skins are not safe for valorizing into food products as well as for animal forages without detoxification of their toxicants. Other toxicological qualities of fruit by-products are scarcely studied.

### 4.3 Banana

According to the FAOSTAT database [22], bananas were the first globally produced fruit in 2022 (Figure 1). During the banana processing, the peel part accounts for about 35%. Hence, this percentage of the banana fruit is wasted throughout the processing unless it can be valorized.

The heavy metals analyzed in banana peels were As, Cd, Cr, Co, and Pb, and their concentrations are summarized in Table 1 [36,44,45]. In all the analyzed reports, the concentrations of the heavy metals are below the range of the hazardous limits [2].

Šeremet et al. [105] studied the cytotoxicity of banana peel extract on cells of tongue epithelium (CAL 27), colon epithelial cells (Caco-2), and liver cells (HepG2) in a range of 0.014–10 mg/mL extract concentration and treatment times of 0.5, 1, and 2 h. They reported that polysaccharides containing banana peel aqueous extract has no cytotoxic/proliferative effect on epithelium (CAL 27) and liver cells (HepG2) at any of the tested concentrations or treatment times, whereas 10 mg/mL of the polysaccharides-free aqueous extract has shown slight proliferative effect in all three treatment times on the colon epithelial cells (Caco-2).

*Fusarium proliferatum* is a fungal species that causes crown rot in banana fruits mainly occurring during infection on postharvest [106]. Studies have shown that fumonisin B1 can contaminate banana peels during postharvest [106,107]. Gomes et al. [65] reviewed pesticide concentrations in bananas. Accordingly, they reported that pesticides such as chlorpyrifos and methiocarb are within the minimum residual limit set by Codex. Moreover, Mohd Zaini et al. [66] summarized the anti-nutrient composition of banana peel processing using different processing conditions (Table 2). From their report, the phytate, alkaloids, oxalate, and glycoside content in the banana processed by fermentation has shown the maximum concentrations compared to the banana processed by microwave drying, boiling, and air and oven drying methods. A large concentration of glycosides was found in banana peel [108]. Cyanogenic glycosides are the main precursor of HCN, which can be converted through hydrolysis. Glycosides are carcinogens and HCN are also toxic substance, which are formed during the chemical reaction of acids with metal cyanides [66]. Furthermore, the banana and papaya peels contain about 900 and 500 mg/kg of tannins [71]. This value could be varied due to different conditions. Hence, careful reduction mechanisms of banana fruit peel contaminants are required during valorization.

### 4.4 Papaya

Papayas have 13.8 million tons of global production in 2022 as shown in Figure 1 [22]. The waste part (by-product) of papaya rind and seeds accounts for 10–20% [33]. Hence, much of the fruit waste can be valorized into valuable products.

Availability of heavy metals such as As, Cd, Cr, Co, Ni, and Pb were reported in papaya fruit by-products, and their concentrations are summarized in Table 1 [45,48,49]. Except for the Cr, which is slightly higher, all the reported heavy metals were below the range of the hazardous limits [2].

Kumar et al. [48] investigated the presence of higher nickel in a mature papaya peel (13.2 mg/kg) than in young peel (7.96 mg/kg), mature seed (7.41 mg/kg), and yang seed (5.99 mg/kg). Moreover, about 7.36 and 6.41 mg/kg of chromium in mature and young peel were determined, respectively. Similarly, the cobalt analyzed in mature seeds and young peels were 0.15 and 0.4 mg/kg, respectively. In another study reported by Vinha et al. [49], the heavy metal such as chromium, cobalt, arsenic, cadmium, nickel, and lead were determined as summarized in Table 1.

The ANFs such as tannins, oxalates, and phytate analyzed in pawpaw (*Carica papaya*) seed flour are presented in Table 2 [76]. Toxicants in papaya seed such as phytates (3.04%), glucosinolates (10%), tannins (6.35%), and isothiocyanate (0.03%) were analyzed as dry weight of defatted seed meal. Compared to other toxicants glucosinolates account for the highest proportion of the fruit seed. Besides, the presence of isothiocyanate in the papaya seed oil implies the thioglucosinolate present in the seed hydrolysis to some degree by the thioglycosidase enzyme [109,110]. Thus, the presence of these toxicants limits the use of papaya seed and its oil for animal or human consumption unless further processing to remove these toxicants could be adopted. Pineapple skin contains larger (597 mg/kg) total toxic metal contents than orange peel, watermelon rind, banana peel, apple pomace, strawberry pomace, and grape pomace, in all of which it is below 50 mg/kg [14].

### 4.5 Watermelon

Watermelon fruits have global production of 100 million tons in 2022 [22], which is the second most highly produced fruit. The watermelon fruit contains a high amount of water (91%) and sugar (6%). From its total weight of analysis, it consists of rind and seeds, which account for 40–45% [111].

Heavy metal concentrations of *Citrullus lanatus* (watermelon) and *Citrullus colocynthis* (*egusi* melon) seeds varieties were reported considering selenium (13–28 mg/kg), cadmium (0.008–0.1 mg/kg), and lead (0.06–0.09 mg/kg). These heavy metal concentrations are below the ADI level (Pb = 0.21–0.25 and Cd = 0.06–0.07 mg/day) developed by FAO/WHO regulations [55].

In a study conducted on pesticide residues (diazinon, dimethoate, and metalaxyl) in flesh and the flesh plus peel (rind) of watermelon samples, the dimethoate was found in higher concentration (diazinon = 2.1 × 10^−5^ mg/kg, dimethoate = 1.95 × 10^−3^ mg/kg, and metalaxyl = 2.9 × 10^−5^ mg/kg) in the rind part. However, this was much lower than the recommended MRL (dimethoate = 2 × 10^−2^ mg/kg) [82]. Hence, properly treated watermelon rind could be the main source of bioactive compound recovery, functional, nutraceutical, and industrial applications [112,113].

Jyothi Lakshmi and Kaul [81] investigated the anti-nutritional quality of watermelon whole meal seeds. They reported that the phytate (9,900 mg/kg), tannin (32 × 10^5^ mg/kg), and oxalate (2,130 mg/kg). These amounts could cause health effects and require pretreatment of the watermelon by-products before consumption or valorization.

### 4.6 Melon

About 28.6 million tons of melon and other related fruit products were produced globally in 2022 (Figure 1). Melon by-products mainly peels and seeds, which account for 58–62% of the raw material, are discarded as residue. These waste parts are rich sources of nutritional qualities, bioactive compounds, and other valuable products [114]. Therefore, many of these fruit by-products could be valorized into valuable products.

Anti-nutritional contaminants such as saponins, oxalates, phytates, and tannins were found in melon by-products, and their concentrations are presented in Table 2 [17]. Other hazardous heavy metals, toxicant organic compounds, mycotoxins, and pesticides/fungicides were scarcely studied on fruit melon by-products. Hence, further studies on these issues are advisable for better valorization.

## 5 Toxicological qualities of fruit berries

Fruit berries contain by-products that can be valorized into many products. The inedible fractions of blueberries, cranberries, raspberries, and strawberries from different literature data were reviewed by De Laurentiis et al. [115]. They reported that fruit berries such as blueberries (9–15%), cranberries (14–17%), raspberries (0%), and strawberries (2–6%) fractions were found. Except for the blueberries and strawberries, other fruit berry by-product toxicity analysis was scarcely studied. Therefore, further investigations on toxicity analysis are required before valorizing them.

Some studies indicated that the berry fruit by-products are susceptible to mycotoxins. In particular, mycotoxins like AOH, monomethyl ether, tentoxin, aflatoxins, and OTA were found in fruit berry by-products [116]. Moreover, some fungicides such as carbendazim and thiophanatemethyl (Table 2) were found beyond the MRL developed by European Food Safety et al. [85], which are carbendazim (0.1 mg/kg) and thiophanatemethyl (0.1 mg/kg).

### 5.1 Blueberries

Shotyk [46] studied the heavy metals in wild blueberries and raspberries considering the metals such as Cd, Cr, and Co, whose amounts are summarized in Table 1. They reported that the raspberries contained slightly lower toxic elements considering the established limit of 0.01 mg/100 g (according to Regulation (EC) No. 396/2005 of the European Parliament and Council).

The chlorpyrifos-methyl concentration (4.27 × 10^−3^ mg/kg) in the blueberry bluecrop variety was reported below the established limit of 0.01 mg/100 g (according to the Regulation (EC) No. 396/2005 of the European Parliament and Council) [67]. Moreover, other blueberries from Serbia contained thiametoxan and azoxytrobin (shown in Table 2) below the abovementioned established limit [68].

### 5.2 Strawberries

The Cr (1.47 × 10^−6^ – 3.41 × 10^−6^ mg/kg) and Ni (2.33 × 10^−6^ – 3.56 × 10^−6^ mg/kg) analyzed in strawberry fruits grown in different mediums containing different minerals were found to be below the maximum daily intake limit (Ni = 0.002 mg/kg and Cr = 1.5 mg/kg) [54].

Shao et al. [53] studied pesticides, phthalates, and heavy metals in strawberries grown in Shanghai, China. Phthalates such as bis-2-ethylhexyl phthalate (DEHP), diisobutyl phthalate (DIBP), and dibutyl phthalate (DBP), as well as heavy metal residues like Pb, Cd, and Ni were detected in strawberry pomace. Moreover, the dominantly detected pesticides were procymidone, acetamiprid, boscalid, and carbendazim and their concentrations are summarized in Table 2. They reported that the pesticides, DEHP, DIBP, and DBP, as well as the lead, cadmium, and nickel, were below the estimated daily intake.

Strawberry press-cake is the main source of ellagitannins and dietary fiber, which are important for human health. Total pesticides (2.143 mg/kg) mainly containing fungicides and insecticides are present in this by-product. The ADI (% ADI) of pesticides in the ellagitannins should be between 0.2 and 4.1% [80]. Hence, this strawberry press-cake is in the acceptable range for a human dose, which is equivalent to 100 g of strawberries.

The MRL of some fungicides such as carbendazim (0.1 mg/kg), thiabendazole (0.05 mg/kg), imazalil (0.05 mg/kg), thiophanatemethyl (0.1 mg/kg), and insecticides such as λ-cyhalothrin (0.01 mg/kg), carbofuran (0.05 mg/kg), formethanate (0.05 mg/kg), and fenoxicarb (0.05 mg/kg) in strawberries was developed by European Food Safety et al. [85]. Except for the carbendazim (0.1 mg/kg) and thiophanatemethyl (0.1 mg/kg) in strawberries, which were reported equivalent to the MRL, all the analyzed fungicides and pesticides were below the MRLS [80]. Hence, since the concentrations of the above fungicides and pesticides can vary from area to area as well as during measurements, their reduction mechanisms is very much required during valorization.

## 6 Toxicological qualities of other fruits by-products

Many other fruit by-products listed below are also produced globally. According to the study reported by De Laurentiis et al. [115], conducted on the amount of fruit purchased and related unavoidable wastes by EU households in 2010, apples (1.2 metric tons), apricots (0.03 metric ton), cherries (0.05 metric ton), and sour cherries (0.01 metric ton) were reported as waste.

Some hazardous metals, mycotoxins, toxicant organic compounds, ANFs, and pesticides/fungicides that are present in fruit by-products were reported. For instance, pesticides such as thiram, chlorpyrifos, and methyl parathion were found in apple and mango fruit peels [9].

### 6.1 Cherry

Cherries have a global production of 2.8 million tons in 2022 that is shown in Figure 1 [22]. However, about 0.05 metric tons of fruits are unavoidable wastes [115]. Hence, many of these by-products can be valorized into many valuable products.

Pesticide residues in sweet Cherry pits analyzed beyond the MRL set by the European Commission, 2005 (0.01 mg/kg for imidacloprid and phosmet) were phosmet (0.023–0.439 mg/kg) and imidacloprid (0.029 mg/kg). However, many of the other types of pesticide residuals were reported below the maximum residual limit (Table 2). The total pesticide residues in this fruit seeds were reported ranging from 0.139 to 2.544 mg/kg [117].

Mateus et al. [117] analyzed the availability of mycotoxins in sweet cherry pits. They reported that none of the mycotoxins from the analyzed nine mycotoxins aflatoxins (AFs), ochratoxin A (OTA), fumonisins (FBs), zearalenone (ZEN), and trichothecenes (T-2) were found in the sweet cherry pits.

The valorization methods of cherry by-products should consider a further analysis of hazardous metals, mycotoxins, pesticides, ANFs, and toxicant organic components, since limited studies have been conducted.

### 6.2 Apple

Apple is the third globally most-produced with 95.8 million tons in 2022 (Figure 1). During the study conducted on the amount of fruit purchased and related unavoidable wastes by EU households in 2010, apples had 1.2 metric tons of unavoidable waste [115]. During the apple processing, its pomace, peel, and seeds are found as by-products.

Some studies have reported that apple peels contain heavy metals such as Cr and Cd. In particular, the heavy metal concentrations (Cu = 3.7, Zn = 4.13, Cr = 2.25, and Cd = 0.002 mg/kg) analyzed in apple peel were below the safety qualification for agricultural products except for chromium (Cr = 0.5 mg/kg) [41]. Moreover, heavy metals (Cd, Cr, Ni, and Pb) found in apple pomace were analyzed below the permissible limits to implicate their non-toxicity during biogas production (Table 1) [37].

Fruit by-products may contain naturally occurring plant toxins like cyanogenic glycosides, which include AMG. In particular, the AMG composition studied in apple seeds (from 1,000 to 4,000 mg/kg) is higher than its pulp fruit juice. Hence, excessive ingestion of this fruit seed can cause sub-acute cyanide poisoning. However, fruit processing methods such as crushing, fermentation, boiling, soaking, and drying help to reduce the cyanide contents in such fruits [57]. Similarly, the naphthaleneacetic acid residue on seven varieties of apple skin was found to be 43.3 mg/kg [61].

From the review reported by Georganas et al. [98], the potential hazards found in apple by-products were AMG, pesticides (e.g., neonicotinoids and arsenic-based pesticides), patulin. The AMG is found in the apple seeds, which can cause acute cyanide poisoning in humans with the consumption of about 800 g of apple pomace. Moreover, the pesticide residues like neonicotinoids and acetamiprid were detected in apple pomace. Fungicides such as thiophanate, carbendazim, and pyrimethanil in apple pomace were detected in apple pomace although the reported amounts were below the toxicity level set by the United States environmental protection agency [118].

Pavicich et al. [59] investigated Alternaria mycotoxins such as AOH, AME, AME 3-sulfate (AME-3-S), altenuene (ALT), tenuazonic acid (TeA), tentoxin (TEN), alternariol 3-glucoside (AOH-3-G), AME 3-glucoside (AME-3-G), and altertoxin-I (ATX-I), alternariol 3-sulfate (AOH-3-S) present in apple by-products (pomace). These by-products passed through different processing (grinding, clarification, centrifugation, and water evaporation) were found to contain AME-3-S, and AME-3-G (Table 2). Alternaria mycotoxins are considered relatively stable and their contamination levels can be reduced during apple concentrate processing listed above.

The ANFs reported in apple fruit peels were oxalates, HCN, alkaloids, and phytates, and their concentrations are summarized in Table 2. The reported value for HCN in this fruit peel is below the threshold value (below 3,500 mg/kg) reported as the safety limit [60].

Pesticides such as boscalid and deltamethrin mainly concentrate in the apple skin rather than in the pulp part. These pesticides can be removed employing thermal processing. However, pesticides like acetamiprid cannot be reduced by drying, wet pasteurization, and frying processes except by using the lyophilization method [58].

The overall analysis of the reported data on the toxicity of apple fruit by-products is healthy for further valorization. However, due to these data varying from production place to place and from measurement to measurement, the valorization method should be done concurrently with toxicity investigations.

### 6.3 Mango

Mangoes and their related fruits have a global production of 59.2 million tons in 2022 (Figure 1). Mango processing has by-products of mainly peel and stone, which account for 45% of the total fresh fruit weight.

de Matuoka e Chiocchetti et al. [45] investigated the heavy metals (Co = 28 and Cr = 0.33 mg/kg) present in mango fruit peels. The reported amounts are above the range of hazard quotient Cr = 0.81–3.18 mg/kg and Co = 0.03–0.09 mg/kg, respectively [2]. Hence, a careful reduction method of these heavy metals is required.

Mango peels contain higher phytic acid (254.8 mg/kg) and oxalate (724 mg/kg) than its pulp part [70]. Moreover, mango seed kernels contain a large amount (153 × 10^3^ mg/kg) of tannins [71]. The ANFs reported in mango fruit peels were oxalates, HCN, alkaloids, and phytates, whose concentrations are summarized in Table 2. The reported HCN concentration in mango fruit peels is below the threshold value (below 3,500 mg/kg) reported as the safety limit [60]. Moreover, the anti-nutritional contaminants present in mango kernel flour were tannins, phytic acid, oxalates, and HCN. The reported concentrations (mg/kg) for tannins, phytic acid, oxalates, and HCN were 1027.4, 1149.8, 213.4, and 0.00, respectively [63]. These ANFs are below the safety limit stated above.

### 6.4 Plum

The plums and sloes together have a global production of 12.4 million tons in 2022 (Figure). According to the study conducted on the amount of fruit purchased and related unavoidable wastes by EU households in 2010, from the 0.58 metric tons of plums and sloes purchased, about 0.04 metric tons were reported as unavoidable waste [115].

Mohammadi-Moghaddam et al. [52] analyzed heavy metals such as As, Cd, Ni, Hg, and Pb in the black plum peel. They reported that the Hg, Cd, and Pb were absent, whereas As and Ni were 1.2 mg/kg and 2.8 mg/kg, respectively. Moreover, Akter et al. [51] reported the heavy metal such as As, Cd, Co, Ni, Pb, and Hg concentrations in plum kernels. They reported that the As and Hg concentrations were <0.1 mg/kg. These heavy metal concentrations were reported as below the tolerable upper intake level recommended by many Food Safety authorities.

Other toxicity reports were scarcely reported. Hence, further studies are required to fully valorize the plum fruit by-products.

### 6.5 Peaches

Peaches and nectarines together have a global production of 26.4 million tons in 2022 as shown in Figure 1. According to the study conducted on the amount of fruit purchased and related unavoidable wastes by EU households in 2010, from the 1.85 metric tons of peaches and nectarines purchased, about 0.16 metric tons were reported as unavoidable waste [115].

The mineral content of peach fruit homogenized with its peel and pulp was analyzed. The heavy metals considered in this study were reported as chromium (Cr = 0.17–1.38 mg/kg) and lead (Pb < 0.10 mg/kg). These results are below the maximum permissible limit value (2.3 mg/kg) for human consumption [50].

Other toxicity properties of the peach fruit by-products are scarcely investigated. Hence, further studies are required for better valorization of the by-products.

### 6.6 Apricot

Apricots have a global production of 3.9 million tons in 2022 (Figure 1). According to the study conducted on the amount of fruit purchased and related unavoidable wastes by EU households in 2010, from the 0.4 metric tons of apricot fruit purchased, about 0.03 metric tons were reported as unavoidable waste [115]. Hence, the unavoidable waste can be valorized into valuable products.

Heavy metals such as cobalt (Co), cadmium (Cd), and lead (Pb) were studied in apricot kernel and pomace. The heavy mineral concentration reported in the apricot kernel and pomace are summarized in Table 1. However, the concentration of lead (Pb) in these by-products is reported as insignificant [42]. The AMG content in apricot seeds (AMG = 52,000 mg/kg) did not induce any effect in rabbit spermatozoa conducted *in vivo*. In particular, the dose of apricot seeds (3.0 mg AMG/kg body weight) on the rabbit spermatozoa parameters did not induce any change [62].

The anti-nutritional contaminants present in apricot kernel flour were tannins, phytic acid, oxalates, and HCN. Moreover, these ANFs were investigated in raw peach fruit kernel flour. The concentrations of these ANFs are presented in Table 2 [63].

The aflatoxins B1 and B2 present in apricot kernels were measured in the range of 0.0017–22.451 mg/kg. In this study, removing the discolored kernels was able to remove 97.3–99.5% of the total aflatoxins [64].

Generally, the apricot by-products have less toxicity although further confirmation tests are important during valorization.

## 7 Current trends on fruit by-product toxicant reduction

**Figure 4:**
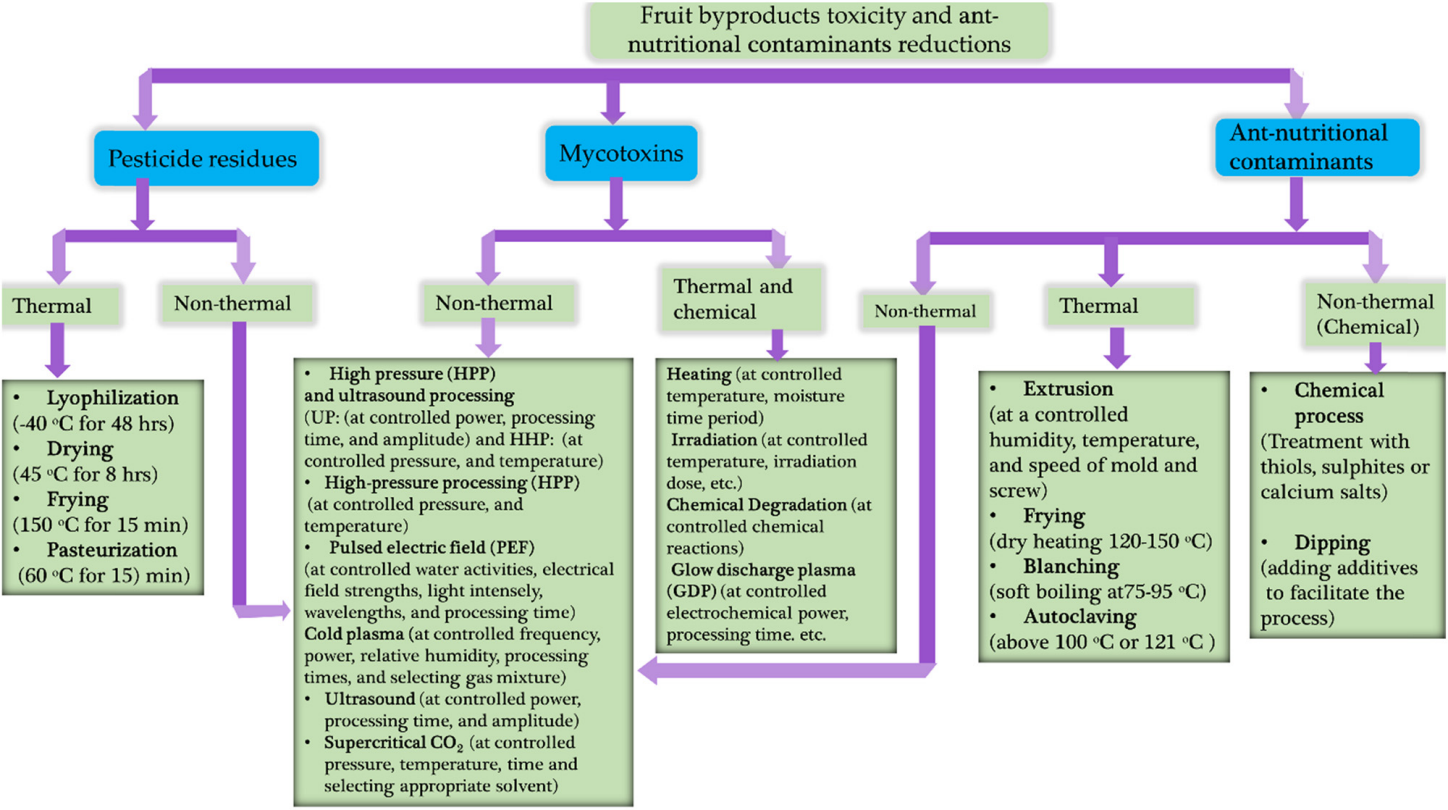
Methods for fruit by-product toxicity reduction.

Several foods worldwide are contaminated by pesticides and mycotoxins due to the pollution of fruits, vegetables, and cereals [119,120,121,122,123,124]. Pesticide reduction constitutes one of the sustainable development goals. Gavahian and Cullen [125] reported on the use of innovative food processing technologies, including high-pressure processing (HPP), pulsed electric fields (PEF), cold plasma (CP), supercritical carbon dioxide, and ultrasound (USN) processing as those with good potential for mycotoxin and pesticide reduction. They depend on processing parameters, the type of pesticide/mycotoxin, and the food matrix. Some of the thermal, chemical, and non-thermal mechanisms of toxicant reduction are depicted in Figure 4.

In the same context, Adebo et al. [126] studied novel non-thermal food processing techniques, particularly HPP, PEF, CP, and USN processing for the decontamination of mycotoxins in food with complete decontamination of mycotoxins in some cases. They also discussed the mechanisms by which reduction/elimination occurs. This takes place through the decomposition of toxins after collision with ions/electrons. Cleavage of bonds, structural degradation of the mycotoxin structure, and cleavage of functional groups are the immediate effects after this decomposition. Other mechanisms include Photolysis/photolytic damage leading to an attack on double bonds and/or heterocyclic moieties in the mycotoxin molecule, dihydroxylation, dehydrogenation, modification of terminal furan ring/alteration, and hydrolysis of lactone ring.

Natural and chemical decontamination of mycotoxins leading to significantly reduced levels in foods with no generation of degradable toxic by-products has been discussed by Agriopoulou et al. [127].

Prevention strategies should also be followed throughout the food production chain. Management should take place before any fungal infestation; the second step of control should be during the period of fungal invasion of plant material and mycotoxin production; and the third step should be initiated when the agricultural products have been identified as heavily contaminated as reported by Čolović et al. [128].

Wang et al. [129] suggested that protective agents, including plant extracts, yeast products, bacteria, peptides, enzymes, H_2_, oligosaccharides, amino acids, adsorbents, vitamins, and selenium could reduce effectively DON-induced organ toxicity.

Fu et al. [130] showed that ZEN exposure resulted in oxidative stress and ferroptosis by glutathione-dependency. Moreover, melatonin supplement through enhanced productions of glutathione peroxidase 4 and glutathione alleviate ZEN-induced abnormalities. Similarly, Zhang et al. [131] discussed the detoxification of AFB1 in ducks’ primary hepatocytes by the key glutathione S-transferase (GST) isozymes.

EOs with broad-range antimicrobial effectiveness, low toxicity, and diverse mechanisms of action have been discussed by Prakash et al. [132] with detailed mechanistic understanding, safety profile, and risk assessment. Singh et al. [133] referred to the use of *Coleus aromaticus* EO, with thymol as the major compound as a natural antimicrobial agent against food-spoilage bacteria and *A. flavus* and AFB1 contamination to extend the shelf-life of food products.

Toxic mechanisms of multiple mycotoxins have been reviewed in the editorial New insight into mycotoxins and bacterial toxins [134].

Lowering of pesticide residue levels in foods can be carried out by washing, blanching, peeling, thermal treatments, alkaline electrolyzed water washing, CP, ultrasonic cleaning, ozone treatment, and enzymatic treatment [135,136]. Significance of precision agricultural practices and integrated pest management (IPM) techniques has been mentioned in the review by Munir et al. [137].

Organic farming methods have been demonstrated to lower the amount of pesticides consumed through food [138]. In addition, IPM techniques – integrating chemical, biological, cultural, and physical approaches to control pests – reduce insecticide applications by 95% while leading to the preservation or increase in crop yields by conserving wild pollinators [139].

Different hormones and internal and external factors influence plant mechanisms ultimately effecting mycotoxin growth, which could be affected by antioxidants and fertilizers available during plantation [140]. For instance, the applications of sodium nitroprusside and melatonin for the distraction of heavy metal stress improve antioxidant response during plantation of crops [141,142].

Dietary fibers and prebiotics recovery from fruits and vegetable wastes and by-products display important biological activities, such as gut microbiota modulation, lowering the glycemic load. These have been mentioned in the review by Pop et al. [143], who also addressed aspects, such as recovery and extraction procedures, characterization, and utilization in different food matrixes.

Proper waste management practices, including waste reduction, safe handling, and appropriate treatment should be followed to alleviate from climate change, environmental degradation, and human health problems. Circularity and sustainable growth are solutions to these problems along with thermophilic microbes in the bioremediation of waste as reported by Najar et al. [144]. These thermophiles emphasize biotechnology and industrial bioprocess progressions toward the build-up of a zero-carbon maintainable bio-economy [145]. Since thermophiles (heat-loving bacteria) can endure extremely high temperatures, they are a major source of various industrial and biotechnological applications with production of enzymes such as amylase, cellulase, protease, xylanases, pullulanases, pectinases, chitinases, esterases, dehydrogenases, and isomerases [146,147].

Thermophilic microbes have been utilized as sources of therapeutic agents, in the food industries, bioremediation, and valorization [148,149].

Moreover, hazardous pollutant remediation from contaminated environments could be carried out by myco-remediation, a green and eco-friendly tool for pollution management as reported by Navina et al. [150], Bhattacharya et al. [151], and Kalia et al. [152]. This is due to the abundance in hyphal network, heavy metal resistance, generation of hydrolytic and degradative enzymes, high surface area to volume ratio, site for metal-binding proteins, high stability and flexibility toward different temperatures and pH. A wide range of contaminants, including pesticides, hydrocarbons, heavy metals, and various synthetic substances can be removed or reduced. Bioremediation refers to the use of organisms for elimination or reduction in pollutants [122].

Three different mechanisms for expunging the environmental pollutants and initiating a balance in the environment are followed by fungi and include bioconversion, biodegradation, and biosorption. Myco-remediation includes the involvement of fungi in the myco-extraction process to remove heavy metals from polluted materials. Fungi accumulate heavy metals and then extraction of heavy metals takes place along with their secure disposal from their biomass [153].

## 8 Novel detoxification techniques

The consumption of foods comprising phytonutrients provides a concurrent anti-oxidation, metal chelation, anti-inflammation, and genes activation related to detoxification. These arrangements are significant in stabilizing the cellular attack by heavy metals and persistent organic pollutants (POPs). As an illustration, the consumption of green tea appears to be a possible dietary line to detoxify heavy metals and POPs. In green tea, the well-investigated flavonoid (epigallocatechin-3-gallate, EGCG) established these protective actions. EGCG triggers the Nrf2-intervened detoxifying and anti-oxidant enzyme like glutathione peroxidase and GST [154]. By arylhydrocarbon receptor suppression and of Nrf2-regulated genes stimulation, Newsome et al. [155] reported that green tea EGCG decreases PCB 126 toxicities. Therefore, green tea consumption seems to be a potential dietary approach to detoxify heavy metals and POPs. On the other hand, it has been stated that quercetin plays a crucial role as a modulator of phase I and phase II detoxifying enzymes. Its molecular structure possesses three conceivable chelating sites, which explicate why it may act as a natural chelator of heavy metals and creates complex with transition metal ions [156]. An animal study displayed that quercetin powerfully regularized the As-induced toxicity in the liver and brain, and Al-induced oxidative harm in the brain [157]. Through its chelation, free radical scavenging, anti-inflammation, and trigger of anti-oxidant detoxification enzymes by upregulation of Nrf2 pathway, García-Niño and Pedraza-Chaverrí [158] confirmed that curcumin defends against heavy metal-induced liver harm. Similar trends reported the same protective effect of curcumin against Cd, As, and cisplatin toxicity [159]. An *in vivo* study concluded that curcumin repressed 2,3,7,8-tetrachlorodibenzo-*p*-dioxin-induced irregular intracellular signaling cascade of cytochrome P450 [157].

Many studies demonstrated the application of novel methods for detoxifications of toxicants in fruit and vegetable by-products. Some of the techniques are low-intensity electrical current and USN applications [160], membrane filtration [161] and application of free chlorine coupled with USN process for pesticide/fungicide residues detoxification [162], microwave, and USN applications for the removal of ANFs [16], and biological detoxification of mycotoxins [163] (Figure 4). Most studies addressed the applications of novel detoxifications for mycotoxins and pesticide/fungicide residues. However, novel technologies for the detoxification of heavy metals (As, Cd, Co, Cr, Ni, Pb, and Hg), toxicant organic compounds, and ANFs are scarcely studied. For instance, Adebo et al. [126] summarized novel non-thermal food processing techniques for mycotoxin reduction, which lead to decomposition of toxins occurred due to collision with ions/electrons by causing cleavage of bonds, degrade structural properties of the mycotoxins, and cleavage of its functional groups. Biological methods such as surface binding by extracellular polymeric substances, degradation by enzyme, and cellular metabolism are potent to reduce toxic metabolites originate from fungi [163]. Moreover, nanotechnology (nanoparticles), PEF, applying plant extracts, and using omics are being alternative solutions to chemical and physical methods for the distraction of mycotoxins in plant products after harvesting [164].

Novel technologies like low-intensity electrical current, membrane filtration, microwave, and USN applications are being more preferable to physicochemical techniques. The physicochemical techniques, are less supportive for mycotoxin detoxification due to causing loss of nutrients, require expensive equipment, and the process can be lengthy [164,165]. Hence, these novel technologies are being applicable for detoxification and preparation of fruit by-products during industrial exploitation.

In plants, consecutive hydroxylation, glycosylation and demethylation of fungal phytotoxins can evade plant cell death and stun the fungal invader [166]. In this line, detoxification of fungal phytotoxins through biotransformation by plants should be a significant plant defensive mechanism against fungal pathogens [166]. By the hydroxylase produced by cruciferous plants like *Brassica napus*, the Destruxin B was detoxified into hydroxydestruxin B [166]. According to Makhuvele et al. [167], ellagic acid and curcumin could avoid the AFB1 metabolism and develop the activity of GST involved in the detoxification of xenobiotics. In addition, numerous glycosyltransferases of mycotoxins existed in plants. A UDP-glucosyltransferase involved in the detoxification of DON was revealed from rice (*Oryza sativa*) [168]. The main detoxification reactions of Alternaria toxins were glycosylation and glucuronidation by plants [168]. In this line, the transformation pathways of Alternaria toxins are (1) glycosylation, (2) hydroxylation, (3) reduction, and (4) methylation and demethylation.

In corn plants, both 15-monoacetoxyscirpenol (15-MAS) and 4,15-diacetoxyscirpenol (4,15-DAS) were, respectively, converted to 15-MAS 3-glucoside and 4,15-DAS 3-glucoside, which are called masked mycotoxins [169]. Regarding citrinin, the chief transformation pathways are (1) decarboxylation: the citrinin was transformed to decarboxycitrinin and (2) oxido-reduction: the citrinin was converted to dihydrocitrinone [169]. In relation to the detoxification of trichothecenes, numerous biotransformation pathways: (1) Glycosylation or glucuronidation, (2) epimerization, (3) de-epoxidation, and (4) hydrolysis [169].

Studies on the removal of pesticide residues from vegetable samples were underscored although limited studies were conducted on fruit by-products [170]. In particular, fungicides and insecticides can be removed by washing with tap and ozone water, ultrasonic cleaning, which could be alternative to other chemical methods using acid/alkali [162]. Moreover, Yang et al. [171] studied the mechanism for the removal of pesticide residues from fresh vegetables by applying free chlorine coupled with USN process. They reported that from 20 to 40 kHz ultrasonic frequency and at 25 mg/L free chlorine concentration, the damage for the vegetable qualities was negligible. In a study reported by Cengiz et al. [27], combined low-intensity electrical current and USN applications were found to be novel techniques to reduce pesticide residues from lettuce samples. This technique was found effective at a current of 1,400 mA and USN frequency of 24 kHz at 10 min. At these combined conditions, the captan, thiamethoxam and metalaxyl residues were reduced by 92.57, 81.99, and 93.09%, respectively.

The hydroxylation usually takes place on pesticide detoxification [172]. For instance, the carbon hydroxylation convoluted in detoxification of chlorsulfuron and triasulfuron by CYP71C6v1 in wheat [173], triasulfuron, fluometuron, linuron, and diuron by CYP71A10 in soybean [174] and pesticide pelargonic acid by CYP72A18 in rice [175]. In addition, the plants Tau and Phi strongly contribute in the pesticides detoxification [176]. In this regard, biophysical and crystallographic valuations exposed that there are at least two ligand–binding sites in GSTs counting the G–site and GSH–binding region [177]. In certain leguminous species, β-alanine substitutes glycine to produce GSH analog homoglutathione (hGSH) [178]. In alfalfa, hGSH replacement of chlorine in atrazine was measured as one of the most essential detoxification pathways [178]. HmGSH conjugates of atrazine and acetochlor are also associated with detoxification of the pesticides in rice [178]. Both hmGSH and hGSH have a nucleophilic thiol group on cysteine residues which can act together with pesticides on their electrophilic metabolites, rendering them detoxified [178]. Additionally, pesticides methylation was described to be crucial for their detoxification in plants [179]. In Medicago sativa, C–methylation of an ethyl group on atrazine has been elucidated [178].

ANFs can be minimized by applying novel detoxification technologies and using fermentation [16,180]. Novel detoxification techniques like microwave heating are being applicable to reduce heat labile ANFs such as phytic acid, trypsin inhibitors, tannins, saponins and oxalate in food products [16] as shown in Figure 4.

## 9 Novel functional foods from fruit by-products free from toxication

Many functional foods can be derived from different food by-products. Fruits contain vitamins, antioxidants, minerals, and dietary fiber.

Globally, 14% of food is lost during harvest. Food loss and waste affect food security and nutrition negatively and significantly lead to greenhouse gas emissions, environmental pollution, degradation of natural ecosystems, and biodiversity loss (https://www.fao.org/policy-support/policy-themes/food-loss-food-waste/en/).

Same for fruit waste where despite the reduction in fruit waste, there is still a need for an improvement in bio-waste utilization.

One such example is pineapple due to its excellent organoleptic quality and nutritional quality and good source of phenolic compounds. Pineapple by-products are used mainly in animal feed and the pharmaceutical industry and constitute 29–40% shell, 9–10% core, 2–6% stem, and 2–4% crown, representing approximately 50% (w/w) of the total weight of the pineapple [77]. The by-products contain dietary fiber, vitamins, minerals, phenolic compounds, and other bioactive compounds [181,182,183,184]. Natural sources of dietary fiber, antioxidants, pectin, enzymes, organic acids, food additives, EOs, etc., through different methods of extractions, purifications, and fermentations could be derived from pineapple waste utilization as reported by Roda and Lambri [185].

Peaches are primarily consumed fresh; however, some peaches are being processed and used as animal feed or fertilizers, or disposed of in landfills from peach by-products (PB) consisting of pulp and peel/skin, constituting 15–28% of the initial weight [186,187]. These by-products could be converted into higher-value byproducts, such as bioplastics at approximately $1,000 per ton of biomass [188]. Peach juice by-products are rich in bioactive compounds, particularly polyphenols, and can produce food additives, antioxidants for pharmaceuticals and cosmetics, and fermentable sugars for bioproducts [189].

Some challenges that need to be taken into account include the composition of the by-products from fruit processing, which depends on the fruit and the stage of processing [186]. Another significant problem might be the underestimation of the real antioxidant capacity and potential of the feedstocks arising from the ineffective characterization of the phenolic content in the extraction of the non-extractable phenolic compounds [177]. The polyphenol profile of both extractable polyphenols and hydrolyzable polyphenols (HPP) from peach by-products and peach peels has been determined [217,218].

Sample preparation and storage conditions are additional factors that need to be carefully designed to prevent perishability and preserve the bioactive components. Reduction in the water content to below 15% is suitable for maintenance of the microbiological quality of dehydrated vegetables while achieving volume reduction [219].

García-Aparicio et al. [220] aimed to assess the PB, generated at the pulp refinement stage for juice concentrate production, as feedstock for a biorefinery for production of fermentable sugars and novel functional products simultaneously. Different conventional and novel enzymatic extraction methods were applied to extract extractable and non-extractable bioactive compounds such as oven drying and freeze drying. They proposed that the solid residue enriched in recalcitrant phenolic compounds and proteins could be used to develop novel functional products for food/feed sectors. Table 3 presents the use of some fruit by-products in the food industry (Adapted from Teshome et al. [28]). Moreover, Table 4 presents medicinal and pharmaceutical use from the exploitation of fruit by-products.

**Table 3:** Uses of some fruit by-products in the food industry

Fruit by-products	Functional foods	References
Apple pomace	Dietary fiber source in baked foods, chicken-meat-based sausages, and yogurt products, stabilizer for oil-water emulsions	[190,191,192]
Avocado by-product and avocado peels	Antioxidants, antimicrobials, and food additives such as colorants, flavorings, thickening agents, and functional beverage formulation	[193,194]
Banana peel	Antioxidant, antibacterial, antifungal activity, reduction in blood sugar, lower cholesterol, and show anti-angiogenic activity and neuro-protective effect, synthesis of bio-inspired silver nanoparticles	[195,196]
Citrus peel	Source of molasses, pectin, oil, and limone, thickener, emulsifier, and stabilizer in many foods, pectin is a suitable polymeric matrix for edible films for active food pack by-product	[197,198]
Grape pomace	Meat and fish derivatives containing grape pomace powders, fiber in bakery products, oil from grape seed	[199,200,201]
Mango peel	Antioxidant and dietary fiber in macaroni, sources of phytochemicals in biscuits, edible films	[202,203,204,205]
Pineapple peel, core and stem	Pineapple peel is a rich source of sugar that can be used as a nutrient in fermentation processes, core can be used in pineapple juice concentrates, vinegar, and wine production, bromelain enzyme extracted from the pineapple stem used as a meat tenderizer, bread dough improver	[206,207]

**Table 4:** Medicinal and pharmaceutical used from the exploitation of fruit by-products

Fruit by-products	Medicinal and pharmaceutical exploitation	References
Apple peel	Reduces metabolic syndrome and atherogenic progression	[208]
Avocado peel	Inhibitor for the inflammation mediator nitric oxide by a possible reduction of free radicals during inflammation, anticancer, antidiabetic, and antihypertensive effects	[103,209]
Banana peel	Antioxidant, antibacterial, antifungal activity, reduce blood sugar, lower cholesterol, and show anti-angiogenic activity and neuro-protective effect, silver nanoparticles, which are used as antimicrobials to pathogenic fungi	[195,210]
Citrus pulp and seed	d-limonene showing a therapeutic effect on lung cancer in mice and breast cancer in mice and rats	[210,211]
Mango	Anti-inflammatory and antioxidative properties during *in vivo* studies related to obesity, diabetes, cardiovascular disease, and skin cancer, reduction of carcinogenesis	[212,213,214]
Peach kernel	Phenols, carotenoids, and cyanogenic glycosides of peach kernel possess antidiabetic, antioxidative, and anti-aging properties	[215]

Adapted from Teshome et al. [28].

## 10 Conclusion

Many of the prioritized fruit by-products have lower toxicity levels, which are analyzed in terms of heavy metals (As, Cd, Co, Cr, Ni, Pb, and Hg), mycotoxins, toxicant organic compounds, ANFs, and pesticide/fungicide residues. However, for full valorization of these by-products, pre-treatment mechanisms should be applied to reduce their toxicity. A holistic revalorization of these by-products with regard to major components, such as fermentable sugars, and value-added components such as the phenolic compounds is required. Details regarding composition, bioactive compounds profile (free and bound compounds), antioxidant capacity, and the impact of the drying process are essential for the development of processes and technologies for their reuse, and targeting of industrial sectors for their exploitation. Mycotoxin detoxification and pesticide reduction mechanisms and control strategies have been discussed as being highly beneficial for the development of food safety and security. The heavy metals, mycotoxins, toxicant organic compounds, anti-nutritional contaminants, and fungicide/pesticide residues of some by-products of fruits prioritized in our study were scarcely studied, Hence, toxicity analysis of each of these fruit by-products demands further investigations.
